# A summary index for antimicrobial resistance in food animals in the Netherlands

**DOI:** 10.1186/s12917-017-1216-z

**Published:** 2017-10-24

**Authors:** Arie H. Havelaar, Haitske Graveland, Jan van de Kassteele, Tizza P. Zomer, Kees Veldman, Martijn Bouwknegt

**Affiliations:** 1 National Institute for Public Health and the Environment, PO Box 1, 3720 BA Bilthoven, the Netherlands; 2 Utrecht University, Faculty of Veterinary Medicine, PO Box 80175, 3508 TD Utrecht, the Netherlands; 3 University of Florida, Emerging Pathogens Institute and Animal Sciences Department, PO Box 100009, Gainesville, FL 32610 USA; 4 Central Veterinary Institute part of Wageningen UR, PO Box 65, 8200 AB Lelystad, the Netherlands

## Abstract

**Background:**

The Dutch government has set targets for reduction of antimicrobial usage in food animals, stipulating a 50% reduction in usage (on a weight basis) in 2013 as compared to 2009 and a 70% decrease in 2015. A monitoring program has been instituted to evaluate the impact on antimicrobial resistance (AMR). The Dutch Ministry of Public Health Welfare and Sports has expressed the need for a summary index to present the results of the monitoring data concisely to policy makers.

**Methods:**

We use data on AMR in bacteria from randomly collected samples from broiler chickens, fattening pigs, veal calves and dairy cows. *Escherichia coli* was selected for resistance monitoring because they are intrinsically susceptible to the antibiotics included in the test panel (ciprofloxacin, cefotaxime, tetracycline and ampicillin) and they are present in all samples, which facilitates proper randomization and trend analysis. The AMR summary index was calculated for each animal species as a weighted average over the four antibiotics, taking into account their clinical relevance. Weights were obtained by conjoint analysis, a pairwise comparison study involving infectious diseases professionals with clinical and public health backgrounds, with data analysis by conditional logistic regression. The AMR summary index was then computed by Monte Carlo simulation, accounting for sampling and regression uncertainty.

**Results:**

The highest weights (0.35) were given to ciprofloxacin and cefotaxime followed by ampicillin (0.23) and tetracycline (0.07). Throughout the years, the AMR index was highest in broiler chickens, followed by pigs and veal calves, while the lowest values were consistently recorded in dairy cows. In all animal species, the index in 2014 was significantly lower than in 2009.

**Conclusions:**

We demonstrate that high-dimensional data on surveillance of antimicrobial resistance can be summarized in an index for evaluating trends between and within food animal species by a process involving decision makers and scientists to select and weight the most relevant antibiotics.

## Introduction

**Fig. 1:**
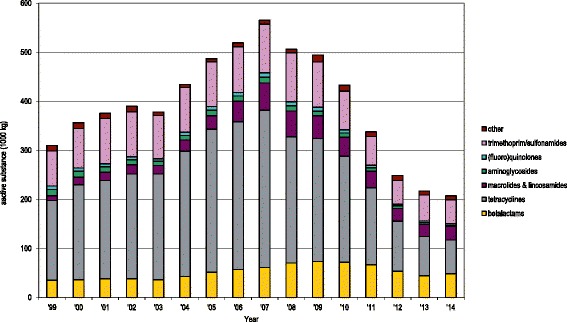
Antimicrobial veterinary medicinal product sales the Netherlands, 1999–2014 in kg (thousands). Published with permission of the Netherlands Veterinary Medicine Authority [1]

Historically, antimicrobial usage in food animals in the Netherlands was high compared to many other industrialized countries [1]. This high usage caused increasing concern because of increasing trends in antimicrobial resistance (AMR) among pathogens and commensal bacteria isolated from food animals. The Dutch government has set targets for reduction of antimicrobial usage in food animals, stipulating a 50% reduction in usage (on a weight basis) in 2013 as compared to 2009 and a 70% decrease in 2015 compared to 2009. In order to achieve this goal, benchmark thresholds for veterinary antimicrobial use on individual livestock farms were decided upon in 2011 by the Netherlands Veterinary Medicines Authority [2]. Antimicrobial usage and AMR are annually reported in the MARAN series of reports, a joint publication by the Central Veterinary Institute, the Netherlands Veterinary Medicines Authority, Utrecht University, the Dutch Food and Consumer Safety Authority, and the National Institute for Public Health and the Environment. Indeed, antimicrobial usage in food animals has decreased from 495 t active substance in 2009 to 217 t in 2013, a decrease of 56% and meeting the target set by government. The decrease was observed in all major food animal species. In 2014, the decrease levelled off, and total sales were 58% below the level of 2009 [1]. Figure 1 shows the time trend of sales of antimicrobial licensed for therapeutic use in the Netherlands, by pharmacotherapeutic group. Sales data show the largest decrease for tetracyclines (73% reduction from 2009 to 2014), although it still was the most used group in 2014. Penicillins (betal-actams) were the second most applied group, sales being reduced 34% between 2009 and 2014.

To evaluate the impact of the reduction in antimicrobial usage on antimicrobial resistance in food animals, a monitoring program has been instituted, in which several pathogenic bacteria (including *Salmonella enterica*, *Campylobacter* spp. and Shiga-toxin producing *Escherichia coli*) and commensal bacteria (including *Escherichia coli* and *Enterococcus* spp.) are included. Food animals as well as foods of animal and non-animal origin are sampled and isolates are subjected to resistance testing by several laboratories, using standardized protocols [3].

The MARAN series of reports offer a comprehensive overview of all AMR monitoring data. However, the level of detail in these reports is difficult to communicate to policy makers and Parliament. Therefore, the Dutch Ministry of Public Health Welfare and Sports has expressed the need for a summary index of antimicrobial resistance in food, in order to report the impact of reducing antibiotics use in food animals on AMR in food-related isolates. Such an index should be presented annually, starting with data from 2009; the base year for the Dutch reduction policy for antibiotics use.

To our knowledge, such a summary index has not been presented before. Aggregating data on antimicrobial resistance requires many choices to be made. These choices involve both scientific and policy aspects. Therefore, an interactive process involving different stakeholders was needed to arrive at a result that is useful for decision-making. This paper describes the construction of such a summary index, documenting the available data, the process to make scientific and policy choices that needed to be made for data aggregation, and presents results up to 2014.

## Methods

### Selection of micro-organisms and antibiotics

A workshop involving policy makers, scientists, veterinarians and medical doctors defined priorities for selection of micro-organisms and antibiotics, and evaluated the currently available monitoring systems. A preliminary selection of the priority micro-organisms and antimicrobial agents was based on an earlier published risk profile [4]. The selection included critically important antibiotics as defined by the World Health Organization and the World Organization for Animal Health (3rd/4th generation cephalosporins and fluoroquinolones), as well as compounds with high usage in the Netherlands (tetracyclines and penicillins), see Table 1. The workshop also considered criteria for the evaluation of surveillance programs [5], and selected the following as most important for the summary index on AMR:

- Continuity (C): Are data collected continuously and in the same way from 2009 to 2013, and will the system be continued in future?
- Representativeness (R): Are the detected cases representative for the target population (healthy animals)?
- Simplicity (E): Have procedures and data collection been restricted to the necessary minimum, and is there a legal basis?
- Data quality (D): Are the data accurate and complete, i.e. are the analyses carried out by accredited laboratories using harmonized protocols?
- Sensitivity (S): Is the sample size sufficient to demonstrate trends (i.e. > = 20 isolates per serovar of species per year).

**Table 1:** Selection of the most relevant combinations of micro-organisms and classes of antimicrobial agents [4]

Class	Antibiotic	*Salmonella* spp.	*Campylobacter* spp.	*E. coli*
**Quinolones**	Ciprofloxacin	X	X	X
**Cephalosporines**	Cefotaxime	X	–	X
**Macrolides**	Erythromycin	–	X	–
***Tetracyclines***	Tetracycline	X	X	X
***Penicillins***	Ampicillin	X	X	X

Clinically important antimicrobial agents are printed in bold, high usage compounds in bold italics

An evaluation of existing AMR monitoring in the Netherlands is presented in Table 2. Based on this evaluation, it was decided to base the AMR index on monitoring of resistance in (commensal) *E. coli* in four species of food animals (broiler chickens, pigs, veal calves and dairy cattle) against four antimicrobial agents (Table 1).

**Table 2:** Evaluation of monitoring of AMR in the Netherlands for implementation in an AMR summary index^1^

Class	Antibiotic	Broiler chickens	Laying hens	Fattening pigs	Veal calves	Dairy cattle
*Salmonella* spp.						
Quinolones	Ciprofloxacin	C-EDS	C-EDS	C-EDS	–	C-EDS
Cephalosporins	Cefotaxime	C-EDS	C-EDS	C-EDS	–	C-EDS
Tetracyclins	Tetracycline	C-EDS	C-EDS	C-EDS	–	C-EDS
Penicillins	Ampicillin	C-EDS	C-EDS	C-EDS	–	C-EDS
*Campylobacter* spp.						
Quinolones	Ciprofloxacin	CREDS	–	-REDS	-REDS	-REDS
Cephalosporins	Cefotaxime	CREDS	–	-REDS	-REDS	-REDS
Tetracyclins	Tetracycline	CREDS	–	-REDS	-REDS	-REDS
Penicillins	Ampicillin	CREDS	–	-REDS	-REDS	-REDS
*E. coli*						
Quinolones	Ciprofloxacin	CREDS	–	CREDS	CREDS	CREDS
Cephalosporins	Cefotaxime	CREDS	–	CREDS	CREDS	CREDS
Tetracyclins	Tetracycline	CREDS	–	CREDS	CREDS	CREDS
Penicillins	Ampicillin	CREDS	–	CREDS	CREDS	CREDS

^1^ *C* Continuity, *R* Representativeness, *E* Simplicity, *D* Data quality, *S* Sensitivity

### Monitoring of AMR in commensal *E. coli* in food animals

The annual monitoring of AMR was performed according to guidelines of the European Food Safety Authority [3] and regulations of the European Union [6] and is described in more detail below.

#### Sampling

Each year, a minimum of 300 samples per animal species from broiler chickens, fattening pigs, veal calves and dairy cows were collected randomly by the Dutch Food and Consumer Safety Authority from a unique epidemiological unit (farm, flock or group of animals). Samples were stored at 2–8 °C and sent to CVI within 48 h after collection for further analysis. Caecal samples of broiler chickens and faecal (or caecal)^1^ samples of fattening pigs were obtained at slaughter during the whole period of the monitoring. Pooled faecal samples of veal calves were collected at farms until the end of 2011 and from 2012 and onwards individual faecal (or caecal)^1^ samples were taken at slaughterhouses. Faecal samples of dairy cows were collected at slaughter (in 2010 and 2011) or as pooled or individual samples at farms (in 2009 and from 2012 onwards). The number of samples tested annually was sufficient to obtain more than 250 *E. coli* isolates per animal species per year which is in compliance with EFSA guidelines (minimum of 170 isolates per animal species). According to the EFSA guidelines, this sample size allows to detect a change of 15% in the situation of widespread resistance (50% proportion of resistance) and to detect an increase of 5% in the situation of few pre-existing resistant isolates (0.1% proportion of resistance). The total set of selected isolates is intended to represent the *E. coli* population of each animal species of the entire country in a given year, and does not provide information on other factors such as seasonality and spatial variation.

#### Isolation, identification and susceptibility testing of E. Coli

Samples were processed in the laboratory on the day of arrival. From each sample, a 10% *w*/*v* suspension was prepared in peptone-glycerol medium. Suspensions were stored at −20 °C in labelled aliquots of 3 ml each pending analysis. On each test day, batches of suspensions were taken from the freezer and thawed at room temperature. Subsequently, 10 μl of each suspension was inoculated directly on MacConkey agar according to EFSA guidelines. MacConkey agar plates were incubated aerobically 16–20 h at 37 °C. The next day, one (red-purple) colony typical for *E. coli*, was randomly picked from the MacConkey agar plate and pure cultured on a blood agar plate. Blood agar plates were incubated aerobically for 16–20 h at 37 °C. Presumed *E. coli* cultures were identified with MALDITOFF. Antimicrobial susceptibility testing of each confirmed *E. coli* isolate against ciprofloxacin, cefotaxime, tetracycline and ampicillin was performed according to ISO guidelines [7] on commercially available antimicrobial panels in compliance with EU regulations [6]. The interpretation of the results was performed using epidemiological cut-off values set by EUCAST.^2^

### Data aggregation

Different methods of aggregating these data were explored. Resistance to critically important antimicrobials was less prevalent than to high usage antimicrobials. A simple unweighted average trend will be dominated by the pattern of resistance in high usage antimicrobials and was not preferred. The summary index was therefore calculated for each animal species as a weighted average over the four antibiotics, taking into account their clinical relevance:

$$ {AMR}_j=\sum_{i=1}^4{w}_i\times {p}_{i,j} $$

with *w*_*i*_ the weight for antimicrobial agent *i* and *p*_*i,j*_ the prevalence of resistance in year *j* (2009–2014) against antimicrobial agent *i*.

Weights were designed to reflect the clinical relevance of the antimicrobial agents and were obtained by conjoint analysis, involving medical microbiologists, epidemiologists, AMR researchers, pharmacologists and clinicians from primary, secondary and tertiary care, who were asked to complete an online questionnaire. The questionnaire was developed in the QuestBack® software (www.questback.com/nl/).Respondents were asked to make pairwise comparisons by answering the following question:

“Consider two patients with the same condition and history (immunological profile, underlying diseases, previous use of antimicrobial agents etc.). Both patients have a bloodstream infection with a (plasmid-related) resistant *Escherichia coli*. One patient is infected by a bacterium with resistance profile A and the other patient with profile B. Which of these patients has a higher probability of recovery?”

Of the 2^4^ = 16 possible profiles, two (sensitive or resistant against all four agents) were omitted from the pairwise comparisons because they were by definition most, resp. least favourable and four were omitted as a combination of resistance to cefotaxime and sensitivity to ampicillin is never observed in practice. Furthermore, all comparisons in which one resistance profile majorized the other (e.g. resistant against both ciprofloxacin and cefotaxime vs. resistant to only ciprofloxacin) were excluded. This resulted in 24 pairwise comparisons per expert. These were randomized both with regard to the order of comparisons and which profile was defined as A or B. To check consistency of the experts’ answers, a random set of six comparisons was added at random positions in the set, with assignment of resistance profiles as A and B reversed. In this way, five random sets were generated and each expert was randomly assigned to one of these sets.

The outcomes were analysed using conditional logistic regression in the statistical software R [8].The four antibiotics were included in the model as explanatory variables, while conditioning on the person-profile combination. The resulting coefficients were transformed into weights by dividing them by the sum of the coefficients. Confidence intervals were obtained by Monte Carlo simulation, where 1000 samples were generated based on the estimated coefficients and their covariance and assuming a multivariate Normal distribution. Uncertainty in the prevalence of resistance among *E. coli* isolates was quantified by a Beta distribution: *p*_*i*, *j*_~*Beta*(*r*_*i*, *j*_ + 1, *s*_*i*, *j*_ + 1) where r_*i,j*_ is the number of resistant isolates against antimicrobial agent *i* in year *j*, and s_*i,j*_ the number of sensitive isolates against antimicrobial agent *i* in year *j*. The weighted average was computed by Monte Carlo simulation to account for both the uncertainty in clinical relevance and prevalence of AMR, using @RISK (Palisade Corporation, Ithaca, NY), an add-in to Microsoft Excel (Redmond, WA).

## Results

**Fig. 2:**
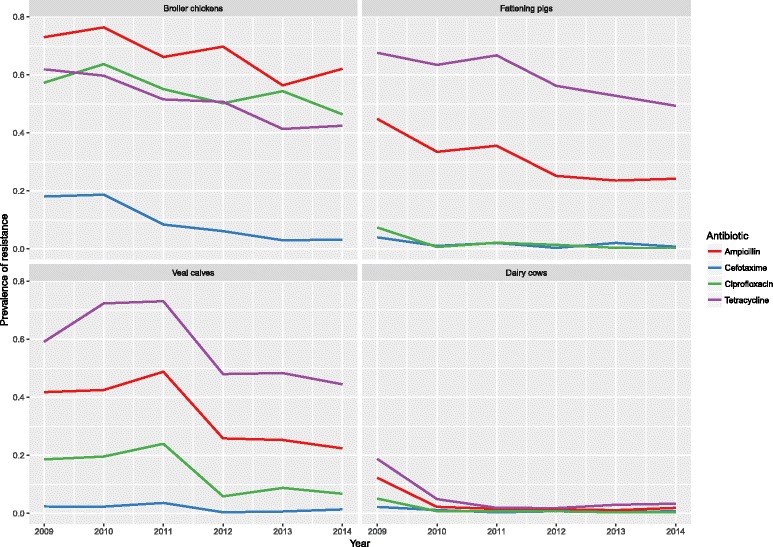
Resistance against antimicrobial agents in *E. coli* isolates from food animals in the Netherlands, 2009–2014

Figure 2 shows the monitoring results from 2009 to 2014 (detailed data in Annex 1). In general, resistance in *E. coli* showed a tendency to decrease in all animal species against all antimicrobials. In all animals except broiler chickens, the prevalence of resistance was Tet > Amp > Cip > Cef. In broiler chickens, a different pattern was observed with Amp > Tet ≈ Cip > Cef.

Twenty-five experts were invited to participate in the pairwise comparisons, and 17 on-line questionnaires were completed (response rate 68%). Only the profession of the non-responders was known, with an equal distribution of non-responders among the four professional groups. Of 17 respondents, 7 were medical microbiologists, 5 clinicians, 2 veterinarians, 1 pharmacologist, 1 AMR researcher/epidemiologist, and 1 a public health physician. Of the 17 respondents, 15 provided consistent answers for the pairwise comparisons that were included twice (but in reverse order) in the set. This implies that the null hypothesis of random completion of the questions was rejected (*p* < 0.0001).

Table 3 shows the utility weights for the four antibiotics. The highest weights were given to ciprofloxacin and cefotaxime (both 0.35) and these did not differ significantly. The weight of ampicillin was significantly lower (0.23) compared to these two, whereas tetracycline was assigned the lowest weight (0.07).

**Table 3:** Weights of antibiotics based on pairwise comparison of resistance profiles

Antibiotic	Mean weight (95% CI)
Ciprofloxacin	0.350 (0.316–0.389)
Cefotaxime	0.350 (0.288–0.416)
Tetracycline	0.068 (0.010–0.116)
Ampicillin	0.231 (0.176–0.292)

**Fig. 3:**
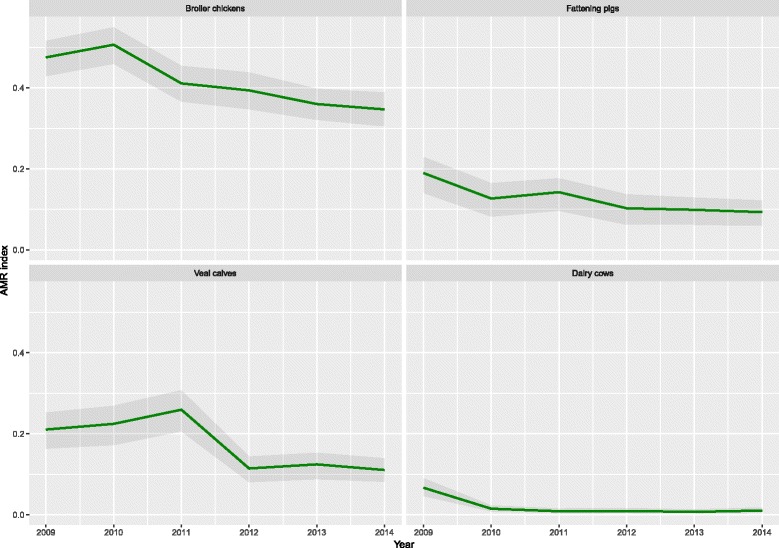
Weighted average resistance (AMR index) against antimicrobial agents in *E. coli* isolates from food animals in the Netherlands, 2009–2014

These weights were combined with the AMR data to calculate a weighted average resistance, see Fig. 3. The graphs confirm a decreasing trend in the weighted resistance prevalence (“AMR summary index”), but the time trends differ between animal species. In broiler chickens, the index was consistently higher than in the other animal species. A slight increase in the AMR index in 2010 was followed by a gradual decrease until 2014. The index in 2014 was significantly lower than in 2010 and 2009 but continued to be at a relatively high level compared to the other animal species. Resistance in fattening pigs decreased steadily over the years and the index in 2014 was significantly different from 2009. Resistance levels in veal calves were slightly higher than in pigs. In veal calves, the index increased between 2009 and 2011, followed by a decline in 2012 and stabilization in later years. The index in 2012–2014 was significantly lower than in 2009–2011. Resistance in dairy cows was significantly lower than in any of the other animal species tested, with a significant decrease of the index to very low levels in 2010, compared to 2009 and then stabilizing at this low level (Fig. 3).

## Discussion

Reducing AMR in food animals is a key policy objective in many countries around the world to control the spread and exposure of humans and animals to resistant pathogens. In the Netherlands, the recent discovery of significant reservoirs of antimicrobial-resistant pathogens such as methicillin-resistant *Staphylococcus aureus* (MRSA) and extended spectrum beta-lactamase-producing bacteria (ESBL) in farm animals, with potential public health implications, combined with an increasing lack of confidence of the public in intensive livestock industries, and discrepancy between the very low antimicrobial use in humans and high use in animals, resulted in intensive collaboration between the government, veterinary professional organizations and important stakeholders within the livestock sector. A combination of compulsory and voluntary actions with clear reduction goals resulted in a 56% reduction in antimicrobial use in farm animals [9]. This study shows that active monitoring of resistance among commensal bacteria in food animals can inform about the impact of such policies on AMR, and that detailed data can be aggregated to summary indices that are meaningful for policy makers. This aggregation included a novel approach to weigh the observed prevalence against different antibiotics for their clinical relevance. We used a pairwise ranking method to obtain weights of the different antimicrobial agents for construction of a pooled estimate. Expert’s answers were highly consistent, indicating overall agreement between different professional groups. The majority of respondents consisted of medical microbiologists and clinicians, of which we knew they were also involved in research. One of two pharmacologists that were invited to participate, responded. Group sizes were too small to allow formal statistical evaluation of differences between respondent groups.

The results suggest that the policy to reduce antimicrobial usage in food animals was effective in reducing the resistance of commensal bacteria. A recent analysis of the association between antimicrobial usage and acquired antimicrobial resistance in *E. coli* in the Netherlands concluded that “drug use history and co-selection of resistance are key elements for perpetuation of resistance” Furthermore, it was concluded that “recent Dutch policies aimed at reducing total use of antimicrobials have decreased *E. coli* resistance in the pig and veal calf production sectors while the impact on the dairy cattle and poultry sectors is less clear” [10].

Indicator or sentinel organisms such as *E. coli* are included in EFSA based AMR-surveillance programs because they are intrinsically susceptible to the antibiotics included in the test panel and they are present in all samples, which facilitates proper randomization and trend analysis. Therefore, trends in AMR-patterns observed may more truly reflect the antibiotic use patterns in the animals the strains are derived from. The association of these patterns with those of food-borne pathogens such as *Salmonella* is not one-in-one, given the fact that *Salmonella* is not present in all animals and all farms and in *Salmonella* trends should be analyzed by serotype. Furthermore, trends in gram-positive bacteria may differ from those in gram-negative bacteria. Still, in the Netherlands, the trends observed in *E. coli*, are predictive of those in food-borne pathogens [1].

The available monitoring data were not fully systematic and the sampling protocol changed over time, to concur with EFSA guidelines that were published in 2012. Therefore, observed trends in AMR among bacteria in food animals in the Netherlands need to be interpreted with care. Nevertheless, our method allows for comprehensive presentation of collected data to a lay public.

## Conclusion

We demonstrate that high-dimensional data on surveillance of antimicrobial resistance can be summarized in an index for evaluating trends between and within food animal species by a process involving decision makers and scientists to select and weight the most relevant antibiotics.

## Abbreviations

AMR: Antimicrobial resistance

## Footnotes

1 Due to the implementation of EU regulations faecal samples of fattening pigs and veal calves were substituted by caecal samples starting from 2014 and onwards.

2
http://www.eucast.org/clinical_breakpoints/
